# Implementing national antimicrobial consumption in Latin America and the Caribbean: opportunities and lessons learned

**DOI:** 10.1017/S0950268824001237

**Published:** 2025-01-14

**Authors:** Gustavo Horacio Marin, Lucia Giangreco, Paola Lichtenberger, Cristian Dorati, Perla Mordujovich-Buschiazzo, Robin Rojas-Cortés, Pilar Ramón-Pardo, Danini Marín, José Luis Castro

**Affiliations:** 1 CUFAR- Faculty of Medicine, National University of La Plata, La Plata, Argentina; 2 Miller School of Medicine, University of Miami, Miami, USA; 3 Pan American Health Organization, Washington, DC, USA.

**Keywords:** AMC, AMR, antibiotics, antimicrobial, consumption, GLASS, Latin America & Caribbean

## Abstract

Surveillance of antimicrobial consumption (AMC) is essential to anticipate and inform policies and public health decisions to prevent and/or contain antimicrobial resistance (AMR). This manuscript shares the experience on AMC data collection in Latin American & Caribbean (LAC). The WHO GLASS-AMC methodology for AMC surveillance was used for data registration during the period 2019–2022. Focal points belonging to each country were contacted and trained for AMC source of information detection, managing registration tools, and data analysis. Thirteen countries were enrolled with significant heterogeneity in the AMC results (range 2.55–36.26 DID-AMC). This experience reflects the heterogeneity of realities in LAC countries; how each one of the nations selected the best sources to collect AMC data, which were the main problems in applying the WHO-AMC collection tool, and the approach that each country gave to the analysis of its data. Finally, some examples are provided on the use of AMC information in making the best decision-making related to AMR control policies at the national level.

## Introduction

Antimicrobial resistance (AMR) represents a major threat to health with significant global economic and safety implications [1]. Antibiotic misuse, particularly when not clinically indicated, directly contributes to the development of resistance to pathogens [2-5].

Therefore, surveillance of antimicrobial consumption (AMC) and use (AMU) is essential to identify areas in which actions and interventions are needed to optimize prescription and dispensing practices. These data should inform stewardship policies at national, regional, and global levels.

In 2014, the World Health Organization (WHO) released the first global report on antimicrobial resistance, which collected national data on nine bacterial infections and antibiotic combinations of great concern for global health [6]. The report not only highlighted the high levels of antibiotic resistance globally but also the lack of coordination and significant gaps in integrated surveillance, especially in many of the developing countries with an elevated burden of AMR, from where no national data was obtained. In order to narrow the gaps in surveillance, WHO launched the Global Antimicrobial Resistance and Use Surveillance System (GLASS) in 2015, which collects epidemiological, clinical and population-level data. One of the modules of the system, referred to here as GLASS-AMC, provides a common technical basis in a big network for setting up national surveillance systems on AMC producing reliable data to monitor and provide countries` own temporal series of data about antimicrobial consumption [7]. The methodology can be used by all countries regardless of the level of development of a country’s national AMC surveillance system.

While the association between AMR and misuse of antimicrobials is well documented, and the WHO-AMC methodology is widely available, there is little information accessible on antimicrobial use in low-income countries. In 2018, GLASS-AMC presented data results from 2015–2016 on the consumption of systemic antibiotics from 65 countries and areas [8]. In the document, only six out of the 35 countries of the Americas presented data, representing only 17% of the nations compared to 85% representation from Europe and 9% in Africa. Those results prompted to question the reasons behind the low participation of countries in the Americas and Africa and raised concerns for possible limitations to implement the AMC methodology in low to middle-income countries (LMIC) [9].

In the past years, the Pan American Health Organization (PAHO), [Regional Office of WHO for the Americas], with the University Centre of Pharmacology of Argentina [Collaborating Centre on Rational Use of Medicines] (CUFAR by its Spanish acronym), developed a strategy to increase the number of countries in Latin America and the Caribbean that implemented a national AMC data collecting systems [10, 11]. Training was provided to Health authorities in data collection and WHO AMC methodology [12, 13].

This paper describes the experience of implementing the national AMC surveillance systems in 13 countries of Latin America and the Caribbean and discusses the lessons learned, challenges and limitations encountered during the process. The conclusions are intended to support health authorities to adopt a validated surveillance methodology, to be applied according to their local needs; and ultimately, provide the basis for interventions to improve the rational use of antimicrobials.

## Materials and methods

The WHO GLASS-AMC methodology for antimicrobial consumption surveillance provides tools that allow the standardization necessary for comparisons [7, 8], especially time-trend comparisons within each country. Without this standardization, comparing antimicrobial consumption would be impossible due to significant variability in pharmaceutical presentations across diverse national markets and over time [14-16]. There exist disparities in the composition of brand names, the concentration of active ingredients, and the size of the packaging, for example.

*Participating Countries*: Thirteen countries were enrolled in this experience: Argentina, Barbados, Brazil, Chile, Colombia, Costa Rica, Cuba, Guyana, Honduras, Paraguay, Peru, St Kitts & Neves and Trinidad & Tobago. The selection criteria were based on convenience. Countries joined the experience by expressing their intention to be included in it.

*Data Classification Tool*: The tools that enable this standardization of the data include:

- The Anatomical Therapeutic Chemical (ATC) Classification system, established by the WHO Collaborating Centre for Drug Statistics Methodology and the Norwegian Institute of Public Health, allows for the identification and coding of each active pharmaceutical ingredient (API) [17].
- The unit of measurement: Defined Daily Dose (DDD), which permits the standardization of the content of each package. The DDD, determined by a Committee of Experts from the same Collaborating Centre, represents the assumed average maintenance dose per day for a drug used for its main indication in adults. Allows transforming the consumption expressed in the packaging of a specific commercial brand (with a certain composition, formulation, and concentration) into a “number of DDD” of active ingredients consumed.
- And the DID: DDD/1000 inhabitants/day, a standardized measure for consumption at the national level, relates the “number of DDD” consumed to the population that consumed these antimicrobials in a given period of time.

Considering this methodology, the antimicrobial groups prioritized (core group) for analysis were J01, A07AA and P01AB because these are the groups with the greatest worldwide use and therefore suggested by the WHO to stat survey consumption at the national level.:

- J01: Antibacterial for systemic use
- A07AA: Antibiotics for the alimentary tract
- P01AB: Nitroimidazole derivatives for protozoal diseases.

Furthermore, GLASS has developed an Excel-based instrument for the automatic calculation of antimicrobial consumption, the “WHO AMC Template.”

*The Implementation Process of the WHO-GLASS-AMC Methodology:* The process carried out by our team in each country consisted of:

- Two initial virtual meetings in which an introduction to the GLASS-AMC methodology was explained, as well as the benefits of performing AMC surveillance in the country.
- Another meeting to determine the national focal point, the personnel responsible for collecting and uploading data in the WHO AMC Template, and the source of information to be used.
- Two virtual training sessions to train designated personnel on collecting and populating data into the WHO tool.
- Two feedback sessions, one after the initial information was uploaded, and another at the end when all the information was entered into the tool.
- Finally, a concluding meeting, featuring a presentation of the results, analysis of the data, discussion of next steps, and feedback was performed.

Data quality control was conducted throughout the entire process close communication with country representatives. During these quality checks, the personnel responsible for the uploading process sent the template to the CUFAR coordination team. The template was reviewed, and suggestions were made to enhance the data loading process and correct errors.

*Training Process:* The CUFAR team with the PAHO members of each regional office assisted countries in conducting AMC surveillance activities for the past five years. The national teams, assigned by country authorities, were responsible for the identification and contact with the available sources of information, and obtaining and uploading the data into the WHO tool. Training on line course was also developed in the PAHO Health Virtual.

*Source of Information*: The sources of information to obtain AMC data were selected and adapted according to each country’s health information systems. In some of them, the decision was made to obtain the data from the public or private sector or both. Data was also analysed at the community or hospital level whenever possible.

*Period of Study*: This analysis includes national AMC data from nine Latin American countries and four English-speaking Caribbean countries, between 2019 and 2022.

## Results

Thirteen countries in Latin America and the English-speaking Caribbean accepted to participate in the WHO AMC national data collection program: Argentina, Barbados, Brazil, Chile, Colombia, Costa Rica, Cuba, Guyana, Honduras, Paraguay, Peru, St Kits & Nevis, and Trinidad & Tobago.

### Country enrolment

In Latin America and the Caribbean, new tasks are often approached with distrust and great resistance, as they increase the workload of an already saturated system.

Therefore, the Ministries of Health of each country were sensitized and discussions were held on the rationale and advantages of monitoring the consumption of antimicrobials. After this first contact, the authorities decided whether to enrol the country in the AMC data collection process or not. If the country’s responsible person decides to initiate the process, then the CUFAR team initiates the training stage.

It is important to mention that one of the topics mentioned in the first contact between PAHO members and the country’s authorities was focused on marking the difference between knowing the consumptions to calculate the local demand for the next year, a very “utilitarian” way focused on the purchasing needs, versus the possibility of knowing the consumptions and being able to compare them with the consumptions of previous years, with to monitor excesses, abuses or irrational use of each antimicrobial, in terms of international recommendations according to Access, Watch and Reserve antimicrobials WHO classification (AWaRe) [14].

Each country delegated one staff member as the focal point whose responsibility was to lead the process and coordinate the local sources of data.

### Source of information, sectors and level of data obtained

Sources of antimicrobial consumption, health sectors, health care levels represented and coverage percentage over the total national population were variable according to each nation’s administration (Table 1). The CUFAR and the regional PAHO team assisted countries` focal points in selecting their own sources from where information would be extracted. Each country decided which sector (private, public or global) and level of information (total, hospital or ambulatory level) to include.

**Table 1.** Sources of antimicrobial consumption information used in each country, health sectors and healthcare levels represented, and coverage percentage over the total national population

	Source of information	Sector			Nivel			% covered population of total country inhabitants			
		Global	Public	Private	Total	Hospital	Community	2019	2020	2021	2022
Argentina	Local manufacturers & imports (ANMAT)	X			X			100			
Barbados	Imports	X			X			NR	100	NR	NR
Brazil	IQVIA			X			X	NR	100	NR	NR
Chile	Procurement records (CENABAST)		X		X			78	77	77.4	76.8
Colombia	Local manufacturers and imports (SISMED)	X			X			100			
Costa Rica	Procurement records (CCSS)		X		X			91.1	91.8	90.9	92.2
Cuba	Distribution records	X			X			NR	NR	100	
Guyana^a^	Procurement records		X		X			6.3			
Honduras	Distribution records (SESAL)		X		X			75	75	75	75
Paraguay	Procurement records (MSPBS)		X		X			NR	74.5	74.1	NR
	Hospital records		X	X		X		12.3	NR	NR	NR
Perú	Dispensing records (MINSA + EsSalud)		x		x			78			82
Saint Kitts	Procurement records		X		X			NR	NR	NR	100
Nevis	Procurement records		X		X	X	X	NR	NR	100	NR
Trinidad and Tobago^b^	Procurement records		X		X	X	X	8.6			NR

Abbreviation: NR: No reported data; ANMAT: National Administration of Drugs, Food, & Medical Technology of Argentina; CENABAST: Central Supply National Health Services System of Chile; SISMED: Drug Price Information System of Colombia; CCSS: Costa Rican Social Security; SESAL: Secretary of Health of Honduras; MSPBS: Ministry of Public Health & Social Welfare of Paraguay; MINSA: Ministry of Health Perú; EsSalud: Social Health Insurance of Perú.

a For Guyana it was considered region 2.

b Trinidad and Tobago data was limited to Eastern Regional Health Authority (EHRA).

Argentina, Barbados, and Colombia monitored AMC at the national level, including both the public and private sectors (global data), and considering the entire population (100%) [18]. Brazil monitored outpatient consumption, using sales data from private pharmacies obtained through IQVIA; it was considered that potentially the entire population has access to these pharmacies, and therefore, the denominator used for DID calculations was the total population of the country.

In contrast, Chile, Costa Rica, Guyana, Honduras, Paraguay, Peru, Saint Kitts and Nevis, and Trinidad and Tobago monitored public sector consumption, with variable proportions of the population covered (ranging from 6.3% in Guyana and 92% in Costa Rica), due to the particular configurations of the different health systems. In the case of Cuba, although the data corresponds to the public system, they are considered to have global data as they include all antimicrobials distributed in the country. In the case of Chile and Peru, the source of information was their own country’s medicine dispensing database (CENABAST and DIGEMID respectively) [15, 16].

Nevis and Trinidad and Tobago were able to disaggregate consumption, obtaining differentiated data for the community and hospital sectors.

In the case of Paraguay, the evaluation of AMC began in 2019 using hospital data from some sentinel public and private institutions. For 2020, they were able to access the total purchases of antimicrobials made by the Ministry of Public Health and Social Welfare.

### Calculation of DID

The AMC results expressed in DID (DDD/1000 inhabitants/day) showed notable differences among countries [17]. A significant heterogeneity in the results was observed in total in periods from 2019–2022 ranging between 2.55 and 36.26 DID (Table 2 and Table 3). The consumption dropped significantly during the pandemic period. However, the overall AMC among countries gradually decreased over the study period. On average AMC was 14.02 DID in 2019; 11.87 DID in 2020; 10.06 DID in 2021 and 10.70 DID in 2022. This means a global reduction of 23.7% in 4 years.

**Table 2.** Yearly evolution of the total antimicrobial consumption expressed in DID per country

	2019	2020	2021	2022
Argentina	36.26	17.42	17.12	24.74
Barbados	NR	16.01	NR	NR
Brazil	NR	7.06	NR	NR
Chile	6.41	5.67	4.12	5.48
Colombia	20.22	22.75	26.30	18.64
Costa Rica	12.75	8.88	9.17	8.50^d^
Cuba	NR	NR	NR	NR
Guyana (Region 2)	13.13	26.02	14.02	12.47
Honduras	13.05	12.18	9.12	7.12
Paraguay^a^	NR	8.45	9.64	NR
Perú	12.50	8.08	8.81	10.33
Saint Kitts	NR	NR	NR	2.55
Nevis^b^	NR	NR	3.55	NR
Trinidad and Tobago (ERHA)^b^^,^^c^	6.19	6.80	4.61	NR

Abbreviation: NR: No reported data.

a The data presented corresponds to the total consumption (hospital + community) of the public sector (procurement records, MSPBS).

b The data presented corresponds to total consumption (hospital + community).

c Only monitored a specific group of antimicrobials, chosen for convenience.

d There is a potential underestimation of AMC for Costa Rica during the year 2022, as the informatic systems of the CCSS were impacted by a cyberattack during that year.

**Table 3.** Country average AMC expressed in DID (%), according to the therapeutical group, during the period 2019–2022

	Intestinal antiinfectives (A07A)	Tetracyclines (J01A)	Amphenicols (J01B)	Beta-lactams, penicillins (J01C)	Other beta-lactams (J01D)	Sulfonamides and trimethoprim (J01E)	Macrolides (J01F)	Aminoglycosides (J01G)	Quinolones (J01M)	Combinations of antibacterials (J01R)	Other antibacterials (J01X)	Agents against amoebiasis and other protozoal diseases (P01A)	Average total consumption
Argentina	0.16 (0.7)	0.87 (3.6)	0	11.78 (49.3)	1.81 (7.6)	0.66 (2.8)	4.56 (19.1)	0.07 (0.3)	2.03 (8.5)	<0.01 (0.04)	0.54 (2.3)	1.40 (5.9)	23.88 (100)
Barbados^a^	0	4.21 (26.3)	0	5.44 (34.0)	2.69 (16.8)	0.48 (3.0)	1.85 (11.6)	0.09 (0.6)	0.60 (3.8)	0	0.09 (0.6)	0.56 (3.5)	16.01 (100)
Brasil^a^	<0.01 (0.1)	0.47 (6.7)	<0.01 (0.1)	2.14 (30.3)	0.72 (10.2)	0.29 (4.1)	2.24 (31.7)	<0.01 (0.1)	1.18 (16.7)	0	<0.01 (0.1)	0.01 (0.1)	7.06 (100)
Chile	0.09 (1.7)	0.06 (1.1)	0	2.24 (41.3)	0.54 (10)	0.18 (3.3)	1.12 (20.7)	0.05 (0.9)	0.55 (10.2)	0	0.45 (8.3)	0.14 (2.6)	5.42 (100)
Colombia	0.31 (1.4)	1.55 (7.1)	0.02 (0.1)	7.67 (34.9)	2.36 (10.7)	0.76 (3.5)	4.37 (19.9)	0.16 (0.7)	2.23 (10.2)	0	1.57 (7.1)	0.99 (4.5)	21.98 (100)
Costa Rica^b^	<0.01 (0.1)	1.63 (16.6)	0	2.22 (22.6)	1.71 (17.4)	1.59 (16.2)	1.01 (10.3)	0.08 (0.8)	0.23 (2.3)	0	1.09 (11.1)	0.26 (2.6)	9.83 (100)
Guyana (Region 2)	0	0.90 (5.5)	0	6.57 (40.0)	0.85 (5.2)	1.89 (11.5)	3.12 (19.1)	0.37 (2.3)	2.02 (12.3)	0	0.69 (4.2)	0	16.41 (100)
Honduras	0	0.53 (5.1)	0	4.68 (45.1)	0.35 (3.4)	1.74 (16.8)	1.87 (18.0)	0.07 (0.7)	0.78 (7.5)	0	0.10 (1.0)	0.24 (2.3)	10.37 (100)
Paraguay^c^	0	0.02 (0.2)	0	3.45 (38.2)	1.69 (18.7)	0.21 (2.3)	2.30 (25.4)	0.03 (0.3)	1.08 (12.0)	0	0.07 (0.8)	0.18 (2.0)	9.04 (100)
Perú	<0.01 (0.1)	0.75 (7.6)	0.02 (0.2)	3.28 (33.0)	1.17 (11.8)	0.57 (5.7)	1.91 (19.2)	0.22 (2.2)	1.31 (13.2)	0	0.32 (3.2)	0.37 (3.7)	9.93 (100)
Saint Kitts^a^	0.02 (0.8)	0.16 (6.3)	0	0.48 (18.8)	0.60 (23.5)	0.18 (7.1)	0.25 (9.8)	0.03 (1.2)	0.55 (21.6)	0	0.11 (4.3)	0.18 (7.1)	2.55 (100)
Nevis^a^^,^^d^	0	0.22 (6.20)	0	0.95 (26.76)	0.65 (18.31)	0.35 (9.86)	0.62 (17.46)	0.12 (3.38)	0.29 (8.17)	0	0.07 (1.97)	0.28 (7.89)	3.55 (100)
Trinidad and Tobago (ERHA)^d^^,^^e^	NR	NR	NR	2.51 (42.8)	1.58 (26.9)	0.61 (10.4)	0.33 (5.6)	NR	0.84 (14.3)	NR	NR	NR	5.87 (100)

Abbreviation: NR: No reported data.

a The data corresponds to the only year evaluated so far.

b There is a potential underestimation of AMC for Costa Rica during the year 2022, and therefore in the average consumption over the entire period, as the informatic systems of the CCSS were impacted by a cyberattack during that year.

c The data corresponds to the total average consumption (hospital + community) of the public sector, for two years, period 2020–2021, using the procurement records of the MSPBS as a source of information (see Tables 1 and 2).

d The data corresponds to the total consumption (hospital + community).

e Average consumption includes data from three years, the period 2019–2021. Only a limited set of antimicrobials were evaluated, selected for convenience.

However, when focusing on each therapeutic group, it was observed certain differences that there was an overuse of some antimicrobials belonging to the group of cephalosporins and macrolides; specifically, ceftriaxone and azithromycin.

During the period 2019–2022, the average consumption of ceftriaxone in relation to the total average consumption of cephalosporins varies from 0.04 to 87.6% depending on the country. According to the national focal points, there was a lack of supply of 1st generation cephalosporins, which are usually used in pre-surgical prophylaxis. Due to the absence of market offers of cephalothin and other cephalosporin from the same group, ceftriaxone was inappropriately used to replace the first-generation cephalosporin in surgical prophylaxis, increasing in 221.9% its overall consumption along the period of study.

When analysing the global consumption of azithromycin in Latin America and the Caribbean, considering only the seven countries that monitored the four years of the period 2019–2022, there was an increase from 6.55 DID in 2019 to 11.26 in 2020 (71.9%). Although a decrease was recorded in 2021 (11.06 DID) and 2022 (10.36 DID), the values have not recovered/reached back to pre-pandemic levels yet.

### AMC according to AWaRe classification

In terms of the Access, Watch and Reserve WHO classification (AWaRe) [14], an AMC pattern recommended is that at least 60% of the antimicrobials consumed belong to the Access group. Among the participating countries, Argentina, Chile, Colombia, Costa Rica, Honduras and Peru remained every year above that percentage, while Guyana, Kitts & Nevis or Trinidad & Tobago fluctuated. The group “Watch” varied according to the changes observed in the “Access” group, while “Reserve” antimicrobials remained stable in the majority of the countries except for Costa Rica (from 0.005 to 0.01) duplicated its consumption of “Reserve” group from 2019 to 2022.

### Quality controls of reported data

Each country uploaded its own AMC data into the tool and sent it to the CUFAR headquarters for quality control. The most common error detected in these documents was shared by all countries and consisted of loading fixed-doses combination products. For example, in the case of beta-lactams with beta-lactamase inhibitors (BLI), it was common to observe that when uploading the product’s concentration, the sum of the antibiotic concentration with that of the BLI was included. According to the WHO methodology, only the concentration of the beta-lactam should be loaded, omitting that of the BLI, as the DDD is established in this manner. On the other hand, when uploading the concentration of cotrimoxazole or antiretrovirals for HIV, which are combined products of two or more antimicrobials, it is necessary to consider the “unit doses or UD.” This is achieved through the use of an equivalence table that matches the concentration of the product to the concentration of a reference product. This aspect is also related to the established DDDs for combined products [19, 20].

In 90% of the cases, errors were observed in the uploading of package size, active ingredient concentration, and/or specific codes determined by the methodology for registering units for each of the variables mentioned.

Less frequently, in 85% of the countries, there were detected mistakes in the assignment of ATC codes, mainly during the process of loading the products whose code varies according to the route of administration, such as metronidazole, vancomycin or neomycin. Other difficulties were linked to combination products, like beta-lactams with IBL, as these combinations often have their codes distinct from the codes of the individual active ingredients. Additionally, confusion arose in the codes within the cephalosporin group, and in some cases, it was detected that instead of using the number ‘0’ during code loading, the letter ‘o’ was mistakenly entered, resulting in the non-identification of the ATC code by the macro functions of the instrument.

In some countries, difficulties emerged at the time of loading antimicrobials whose concentrations are expressed in mass units (mg or g) or power units (IU). For instance, during the loading of colistin, it is necessary to load the concentration in IU in order to perform calculations, as this is the unit in which the DDD is expressed. In Latin America, it is most common to find the concentration of colistin expressed in milligrams (mg), which requires conversion between units. Additionally, depending on the country and the manufacturer laboratory, the concentration expressed as “mg of colistin” may often refer to milligrams of colistin base activity (CBA) or milligrams of the prodrug sodium colistimethate (SCM), which are not the same (1,000,000 IU of colistin = 34 mg of CBA = 80 mg of SCM). Unfortunately, this is not always clarified on product labels, causing difficulty when loading data to calculate AMC. Even, this confusion has been documented during drug administration due to a lack of clarity in descriptions, resulting in overdoses and reported cases of renal failure and death [21].

Another error seen was the loading of antimicrobials for exclusive veterinary use (e.g., enrofloxacin) and antitumor antibiotics -oncological- (e.g., bleomycin).

In two countries, due to unusually high values when calculating consumption, it was detected that initially, during the loading of products, the process involved working with packages/blister packs. However, at a later stage, when entering the number of units consumed, the number of packages/blister packs was not entered; instead, the minimum dispensing units (tablets) were input. This led to an overestimation of consumption, which fortunately was detected during the validation and quality control stage.

## Discussion

The purpose of this study is to report the experience of implementing data collection on antimicrobial consumption in countries of the American continent, applying a collection tool common to all countries provided by the WHO. In this framework, we will review the experience by discussing the events that occurred at each stage of the process.

### About the source of information

Having accepted the challenge of initiating AMC monitoring, the first task of national AMC surveillance teams is to choose reliable information sources from which data will be gathered. The different sources of information for AMC surveillance, such as import and/or manufacturing data, distribution data, drug prescription data in health services, dispensing records or the antimicrobials used by patients allow different levels of detail and disaggregation.

Although the sources of information closest to the patient in the pharmaceutical value chain provide the most reliable data, closest to reality, it is often more complex and expensive to be obtained. On the contrary, data sources that are further away from the patient, such as production, importation, distribution or purchasing data, although less precise due to the fact that not all manufactured/imported/ distributed/purchased medication is actually used, are more accessible and simpler to work with because the data is usually aggregated and consolidated.

On the other hand, and depending on the political configurations of countries, AMC data can be obtained from two different sectors: the public sector, which includes antimicrobials purchased by the government; and the private sector, comprising antimicrobials acquired by third parties.

Similarly, the data collected can also be determined by the level of care provided by the entities that acquire the antimicrobials: hospital (antimicrobials purchased and dispensed by hospital pharmacies to hospitalized patients) or community (antimicrobials bought and dispensed by community pharmacies). Likewise, data collection can encompass both levels or focus on a single level in the absence of data for the second level. Once more, the countries were able to choose among the three options: either disaggregate the data at the community or hospital level or maintain it as a whole if differentiation is not possible (total level).

Comparing the data sources included in this work among Latin-American & Caribbean countries, with other countries and regions, it could be said that heterogeneity is the common feature observed everywhere. For example, in the 29 countries (27 European Union (EU) Member States and two European Economic Area (EEA) countries – Iceland and Norway) ESAC-Network, results show that countries like Germany reported community level consumption, others like Cyprus reported overall consumption without discrimination, while the majority of the countries reported each level by separated files [22, 23].

The key point is the heterogeneity and complexity of each country’s national pharmaceutical markets and drug distribution systems that were used to assess antimicrobial consumption. In the Latin American and Caribbean countries that have conducted AMC assessments during the 2019–2022 period, various approaches were observed in obtaining consumption data, in terms of the sources used and the possibility of disaggregating the data by levels and sectors.

Countries may acquire antimicrobials from local production sources or may import them from other countries. The global data can be obtained by combining these sources. In this case, it may not be possible to break down consumption by sector (public or private) or level (outpatient or hospital), but there will be 100% coverage, encompassing the country’s total consumption. In Argentina and Colombia, drug manufacturing and import data were used, thus achieving 100% population coverage. Similarly, Barbados assessed AMC for the entire population (100%), using exclusively customs records for imports, as the country lacks local production of antimicrobials. If the antimicrobial distribution or purchasing information comes from the Ministries of Health that situation will exclusively represent consumption from the public sector. The representativeness of these data with respect to the national reality will depend on the country, its health system and the percentage of public sector coverage in relation to the total population. These sources may offer the possibility, eventually, of disaggregating consumption according to level, allowing the amount consumed at the hospital and outpatient level to be known. Countries such as Chile, Costa Rica, Cuba, Guyana, Honduras, Paraguay, Peru, Saint Kitts and Nevis, and Trinidad and Tobago used information on purchases, distribution or dispensing from the Ministry of Health or Social Security, corresponding only to the public sector, with variable coverage percentages. However, in most of these countries, the public or social security sectors represent the majority of the population (e.g., Cuba 100%, Costa Rica 97%, Peru 78%).

Another situation was observed in some countries, that faced difficulties in gathering information from all their geographical areas. The absence of centralized medication registries or bills, insufficient funding for travel and for obtaining copies or scanning of paper registries, the lack of internet access in remote areas and a shortage of human resources to collect the pertinent information became an obstacle to performing AMC surveillance. This was the situation faced by the national authorities of Guyana and Trinidad and Tobago that turned some of these limitations into challenges and decided to perform their evaluation in an escalated manner, starting with some regions of the country. In Guyana, consumption in Region 2 was analysed, while in Trinidad and Tobago, consumption was monitored through the Eastern Regional Health Authority (ERHA) [11]. These health teams not only succeeded in conducting the AMC assessment and reporting their own data but also managed to track four years of information in Guyana (2019–2022) and five in Trinidad and Tobago (2017–2021), analysing antimicrobial consumption behaviour before, during, and after the COVID-19 pandemic. Although it will be a future challenge for these countries to improve access to information sources for broader coverage, these initial assessments laid the groundwork for the methodology, provided valuable information, and allowed these countries to train skilled personnel for future evaluations.

An important aspect to highlight is that, although the improvement of information sources and data accessibility should be future goals in all countries, the majority have managed to develop or utilize their own sources. This advancement has allowed them to break free from dependence on commercial sources like IQVIA (formerly IMS Health), which was the only company available for evaluating AMC at a national level until a few years ago.

Despite the prestige of IQVIA and the quality of data that it provides, it is crucial to consider some aspects. To obtain information, the company makes estimations of volumes sold in retail pharmacies (community), and in certain countries, though not all, hospital pharmacies are also included. These estimations are derived from national sample surveys conducted at various points in the drug distribution channels, considered representative of each country (manufacturers, wholesalers, and/or distributors). Subsequently, using an algorithm developed by IQVIA, projections are made to obtain total sales volumes at the national level. Although it is known that this algorithm includes specific factors for each region and drug distribution channel, precise details and factors are not disclosed due to the company’s exclusive ownership [13]. Therefore, this method of information collection determines that the origin of the data and population coverage varies from country to country, not allowing comparisons between them. Additionally, IQVIA does not provide a defined population number that can be used as a denominator when calculating consumption, and determining this number is often a complicated task.

The use of own sources not only avoids costs associated with information acquisition but also allows for better contextualization of the data, providing greater precision regarding the population using monitored antimicrobials and a more detailed understanding of the data’s origin. This, in turn, results in a more robust analysis of consumption data.

### About applying the WHO data collection instrument

The WHO-GLASS has developed an instrument for collecting AMC data, implemented since 2017 in different countries around the world. It consists of an Excel spreadsheet with associated macro functions, which allow the automatic calculation of consumption for each individual antimicrobial, according to the route of administration [9].

Once the information is uploaded, the tool validates the entered data, calculates the consumption in DDD and DID for each antimicrobial individually according to the route of administration, and exports the results of these calculations to a new workbook. It also enables the generation of an additional file for data export to the GLASS system.

During the validation process, if the tool system detects inconsistencies in the information entered in the three work tabs or certain errors in product load, the software identifies them and reports them as a “bug.”

This instrument is updated annually by the WHO, considering any modifications or new additions to ATC and DDD codes that may have occurred, in accordance with the provisions of the WHO Collaborating Centre for Drug Statistics Methodology in Oslo, the centre responsible for the development and update of the ATC/DDD system [17].

Although many countries in the Region have adopted the GLASS-AMC methodology, they used the tool for their AMC data collection at the local level.

Some other countries have implemented their own data collection systems. For instance, Mexico developed its own tool, designed to calculate the necessary annual antimicrobial purchases. Although the instrument effectively fulfils its original purpose, which is the calculation of the annual demand of antimicrobials in order to foresee the purchase that the country will need for the next period; the instrument does not adjust antimicrobial consumption based on population used, avoiding this way the possibility to obtain the DID. Hence, this tool does not allow AMC comparison between different regions of the country or other countries neither to track consumption over the years.

Similarly, Argentina has also developed its data collection instrument, supported by the European Union fund, which has the possibility of calculating AMC. However, this tool does not allow the export of data to GLASS. To do this, it is necessary to migrate the data to the WHO AMC Template.

Another noteworthy initiative regarding the publication of information related to the use of antimicrobials is the case of Chile. Through the CENABAST (Central Supply of the National Health Services System) website, via its observatory, it is possible to publicly access all purchases of antimicrobials made [15].

Currently, Colombia and Peru share their antimicrobial consumption surveillance data through the GLASS-AMC platform, and the information can be visualized online using the GLASS Dashboard [7]. Honduras has recently signed the compromise to report to the GLASS Dashboard. It is interesting to analyse why even when the countries have their own reports in the GLASS tool, they do not make the commitment to report periodically to the GLASS platform. From this experience, we have detected that although countries’ referents make the effort to collect AMC information, they have a certain reluctance to establish further commitments that they are not sure of being able to fulfil in the future. Likewise, some representatives have expressed that the data obtained is useful for decision-making at a local level and they do not see the advantages of sharing their data at a global level. In this sense, it should be noted that there are countries in which CUFAR and PAHO teams have collaborated and obtained information on AMC at the national level, but nevertheless, they have decided not to communicate their data, an aspect that our team respects and accepts since the first objective of this AMC survey takes local measures aimed at the best use of antimicrobials and the reduction of AMR. However, it is very important to understand that AMR is a global problem, and therefore, we must not face it in isolation. We must understand that knowing AMC in each country has positive externalities for other nations, and therefore, reporting to GLASS-WHO is a way to face this problem together. For this reason, we will continue to take measures in order to show the responsible in each country that sharing its own data does not have a punitive or critical meaning, but rather can be useful to correct and jointly confront the AMR problem.

### About the process of training local teams on national AMC data collection

A crucial step in the national AMC assessment is the formation and training of a local team to identify the best source of information, upload collected data into the WHO AMC Template, and perform subsequent data analysis.

In each Latin American and Caribbean country that carried out their national AMC assessments, key persons named “focal points” were identified. These are individuals or work teams with a specific interest in the containment of AMR and, in particular, the methodology for evaluating AMC. After being identified, these focal points expressed their willingness to receive training.

The training was carried out through a course available on the PAHO Virtual Campus for Public Health (VCPH), “Online training on the WHO methodology for antimicrobial consumption surveillance” [12]. The course, in a self-administered format, allows the participants to access the material at any time of the day, advance through the content at their own pace, and have tutoring provided by CUFAR.

Besides the online resource, the CUFAR team provided training support and technical cooperation to the countries through virtual workshops, at the request of the PAHO national AMC-AMR focal points. Additionally, it offered individualized tutoring and assistance to the national teams to resolve questions, detect errors in data entry, and find solutions to possible obstacles in the AMC implementation process, through virtual or in-person meetings, email, and/or instant messaging. Although it is a task that requires time and dedication, the personalized support online or on-site, together with the person responsible for loading the data into the GLASS instrument, has been highly effective and allowed us to leave installed capacity in each country.

### About the most common errors detected during data collection

Analysing AMC using the WHO methodology and the tool designed for this purpose is not overly complex, but it is by no means a routine task; especially when the tool is used for the first time.

The calculation of national AMC, using total local antimicrobial production plus imports as a source of information, may involve the loading of among 250 to 1,300 different products containing one or more antimicrobials when performing the task for the first time, depending on the country. In the case where the source of information is purchases, distribution, or dispensing data from the public sector, the number of products to be loaded is a little smaller, ranging between 40 and 150. In any case, handling these volumes of information increases the probability of slip-up during the loading process.

In subsequent years, the uploading process becomes easier as not all products need to be reloaded. Instead, it suffices to enter the number of packages consumed for the new year. At this point, the difficulty of the task lies in precisely identifying the products that were not used in this new year and the new products that may have been added. The latter must be loaded into the WHO AMC Template at that time.

Based on the experience obtained with the countries of Latin America and the Caribbean, it was observed that frequent difficulties were related to data loading in the WHO tool and resolving bugs that appeared when executing the macro functions [17, 21].

In 60% of the countries, incomplete or erroneous product descriptions (“label”) were identified. These situations were linked, in some cases, to a lack of information or errors in the data sources, and in other cases, to omissions or mistakes when loading information into the WHO AMC Template. An inaccurate or incomplete description can lead to errors in variable input and, consequently, in consumption calculations. As an example, during the entry of “tobramycin 28 mg capsules,” it was mistakenly assumed to be an oral administration formulation and was entered as such when, in reality, it was a formulation for inhalation (powder capsules for inhalation).

### About the quality control stage

The WHO AMC Template, through its associated macro functions, facilitates a first automatic validation to identify certain errors in data loading. This validation can detect empty cells inadvertently omitted when loading information, or data entered inconsistently with the corresponding column, such as the introduction of alphabetical values in columns that only accept numbers, or codes that do not comply with the methodology (for example, to account for tablets or vials, the code “pcs” for “pieces” must be entered; if the word “pieces,” “tablets” or “vials” is entered, the macro will indicate an error).

Although this initial automatic validation is useful, it does not allow the detection of other common loading errors, as mentioned in the previous section. For instance, if an additional zero or one is mistakenly entered when loading the concentration of a product, the macro will not detect it, leading to an overestimation or underestimation of consumption. To avoid these inconveniences, which can later translate into calculation errors, once the data is loaded into the WHO AMC Template, the countries had the option to share them with the CUFAR team for validation and quality control. Upon receiving the information, an initial manual verification process was carried out in order to identify loading errors not detected by the automatic validation of the macro. Once these errors were identified and analysed, suggestions were provided to the national AMC team, who reviewed and corrected the data load. In this way, with these exchanges, a double objective was achieved: a precise load, without errors, and the strengthening of the technical knowledge necessary to apply the methodology. Once all the data are loaded and validated, the AMC is calculated, and the analysis of the information is performed. This stage represented another critical opportunity to identify potential anomalies. Here, errors arose not only linked to the data loading itself but, for example, to the original information sources used to obtain the data. These errors manifested as excessively small or large DID, or as unjustified jumps in consumption within an expected pattern, especially when there were already some years evaluated.

The data validation and quality control stage turned out to be crucial. Difficulties and errors were identified in all countries, which could be corrected at this time, allowing a more precise calculation of consumption that is closer to reality.

### Current limitations and future challenges in the national evaluation of AMC

As important as it is to highlight the progress achieved in terms of the implementation of national AMC surveillance in the Latin America and Caribbean Regions, it is also important to understand the limitations and challenges encountered. Recognizing that there is still a long way to go prompts us to reflect on building new strategies that allow us to obtain more accurate data, achieve broader data coverage, and have better-trained personnel in data entry while speeding up the process and minimizing errors. By obtaining more accurate and global data, countries will have more tools to make better-informed decisions, develop more effective policies, and achieve better public health results regarding AMR containment.

The variation in the results obtained in this study is explained, at least in part, due to the heterogeneity of the information sources used by different countries. These sources are extremely useful for temporal monitoring within the same country and understanding consumption trends in the evaluated sectors. However, precisely because of their heterogeneity, they do not provide the possibility of making reliable comparisons between countries or reaching regional conclusions with certainty.

Although the difficulties in accessing information sources vary considerably between different countries, possibly due to their diverse realities, it is relevant to mention some identified common obstacles.

To date, only Argentina, Barbados, and Colombia have managed to evaluate global consumption with 100% population coverage. In other countries, although some included a population percentage close to 100%, it is still not possible to access global data. In Argentina and Colombia, regulations require that laboratories report all antimicrobials produced, imported, or distributed, along with the corresponding quantity. Considering the possibility of improving access to information sources, a potential solution could be the implementation of specific legislation in this regard.

To assess global AMC with 100% coverage, data from the private sector is required. Therefore, it will be necessary to find ways to access this data, either through cooperation agreements or the implementation of regulations that mandate the mandatory reporting of production, importation, distribution, or sales by this sector.

At this point, it is also relevant to note that consolidated databases may not always exist, and it may be necessary to develop them. Argentina and Colombia have successfully addressed this challenge by building their own information databases to collect the necessary data for AMC analysis.

In the case of Argentina, the National Administration of Drugs, Food, and Medical Technology (ANMAT) has implemented that all laboratories producing and importing antimicrobials in the country have to annually report their production/importation through a sworn statement process, since 2016. From these individual notifications, ANMAT “builds” the necessary database.

In Colombia, the national team uses two different sources of information, which, when combined, provide all the necessary data for AMC surveillance. These sources are the Sanitary Registrations from the National Institute of Surveillance of Medicines and Food (INVIMA) and the Drug Price Information System (SISMED), through which laboratories must report to the Ministry of Health all drugs sold and their prices, whether domestically manufactured or imported.

For nations using information sources from the public sector, one of the future challenges should be obtaining data from the private sector to achieve a comprehensive assessment of global AMC. Despite the extensive population coverage provided by the public sector and social security in many countries in Latin America and the Caribbean, it is crucial to take into account the private sector to understand its consumption profile. Furthermore, factors such as long distances to public health centres or prolonged waiting times may lead individuals to seek private sector consultation. Another situation that could impact global consumption to varying degrees depending on the country, and not considered when relying solely on public sector sources, is the purchase of antimicrobials without a prescription. On the other hand, the sources used by most countries reflect AMC at the national level without the possibility of disaggregating consumption by states, provinces, regions or zones. This limitation prevents a precise correlation with local realities, which may not always coincide with the country’s overall situation. Evaluating consumption solely at the national level without taking into account regional differences may, for example, mask consumption increases in certain areas if consumption declines in neighbouring regions. It will also be important in the future to obtain disaggregated information regarding the level of care to understand different consumption profiles at the outpatient and hospital levels. So far, only Nevis and Trinidad and Tobago have been able to differentiate consumption by level.

Ultimately, for regional harmonization, there is a need to have unified national databases that systematically group all AMC information to assess global consumption. However, at the same time, it will be necessary to establish mechanisms that allow for the disaggregation of that information by sectors, levels of care, and zones. Accessing global consumption data on one hand and minimum levels of information sources on the other will enable the acquisition of more reliable and robust consumption data, with greater comparability, whether between different zones or different sectors of the healthcare system within the same country or across different countries. All of this will lead to a better interpretation and contextualization of the data.

Another aspect related to information sources, which mainly impacts the countries of the Caribbean Region, is the existence of data in physical format and its lack of consolidation, representing significant obstacles, making access to information difficult or unfeasible due to the magnitude of the work required. Digitization becomes imperative and essential to be able to handle large volumes of information efficiently. Likewise, in relation to the limitations of access to technology, in certain areas of this Region, poor Internet connection can constitute an obstacle, making it difficult, for example, to make video calls that are essential for training and the exchange of experiences, as well as access to training material.

In relation to the formation and training of national teams, a frequent limitation is the lack of “protected time” to learn methodological issues and to carry out the necessary technical training, perform data loading, and conduct the review and the analysis of the information. This is possibly a consequence of the scarcity of human and/or financial resources allocated to the AMR containment, and specifically, to the evaluation of AMC.

Furthermore, it is important to remark that in many countries, work teams have not been formed, and a single person has assumed the responsibility of uploading, verifying, and analysing the data. This can hinder the medium- and long-term continuity of AMC evaluations, due to the turnover or replacement of already trained personnel, generally linked to changes in political-health management. Additionally, working in a team could allow, for instance, one person to input data into the WHO AMC Template while another conducts a critical and thorough verification, which could be beneficial in decreasing errors and slip-ups, and ensuring data quality.

### A final discussion of the work performed

Although international experiences offer valuable lessons, they are seldom entirely transferable to the internal reality of each country [24]. Our experience reflects the heterogeneity of the realities that are evident in the different countries of Latin America and the Caribbean. It will be essential for those countries aspiring to implement national monitoring of AMC to consider the specific situation of their own territory, with all its peculiarities in identifying the appropriate sources of information and the sector and level of data that will be obtained. These elements are crucial for the periodical monitoring and follow-up of the AMC, considering that this information will be essential for the decision-making in health policies at the national level. Even though the conditions may not be ideal, taking the first step in AMC registration is important, even if it seems small, as it will allow for the accumulation of experience. Ultimately, the path is made by walking.

## Conclusions

In order to contain antimicrobial resistance, it is imperative and urgent to prevent the misuse of these medicines. Understanding and analysing antimicrobial consumption patterns enable informed decision-making and the implementation of specific measures, allocating resources strategically to address the most pressing issues.

Implementing antimicrobial consumption surveillance in countries with limited resources carries many challenges, but that does not mean it is an unfeasible task. This manuscript shows the heterogeneity among the Latin American and Caribbean countries but also presents their common limitations and challenges encountered. This paper not only enumerates the difficulties but also presents potential solutions that have emerged from experience. We believe that sharing these perspectives may contribute to a collective knowledge base. We hope that this document can empower professionals and decision-makers in countries with limited resources to pave the way for the successful implementation of national AMC surveillance programs.

## Data Availability

In all the processes, nominal patient data were not requested. All the extracted data correspond to the consultation of primary sources of each country regarding the amounts of antimicrobials consumed during the study period. The protocol of the study was validated by the Pan American Health Organization Ethical Committee (PAHOERC). Ref. No: PA-HOERC.0317.01.
